# Association of lifestyle elements with self-rated wellness and health status in patients with Behcet’s disease

**DOI:** 10.1186/s41927-020-00148-1

**Published:** 2020-09-27

**Authors:** Maryam Masoumi, Reihane Tabaraii, Saeed Shakiba, Mansoureh Shakeri, Abbas Smiley

**Affiliations:** 1 Clinical Research Development Center, Qom University of Medical Sciences, Qom, Iran; 2 School of Medicine, Tehran University of Medical Sciences, Tehran, Iran; 3 Westchester Medical Center, New York Medical College, Valhalla, NY USA; 4 Westchester Medical Center, 100 Woods, Valhalla, NY 10595 USA

**Keywords:** Behcet’s disease, Sleep, Lifestyle, Self-rated wellness and health

## Abstract

**Background:**

Assessment of the association of various lifestyle factors and wellness and health status in patients with Behcet’s disease was the main goal of this study.

**Methods:**

Demographic information, body mass index, smoking habit, mood status, sleep quality, physical activity levels, nutritional data, symptoms, signs, laboratory findings and patient reported outcome (self-rated wellness and health) in 52 patients with Behcet’s disease were collected in this cross-sectional study. A multivariable linear regression model was used to assess the association of self-rated wellness and health status and lifestyle factors, adjusted for age, sex, BMI, major symptoms and signs, as well as laboratory findings.

**Results:**

Female to male ratio was 21/31, and the mean age of participants was 44 years. Mean self-rated wellness and health score was 14.6 out of 20. Oral and genital aphthous, ocular involvement, pathergy, and skin involvement were observed in 100, 52, 92, 36.5, and 9.5% of patients, respectively. The mean values of sleep, mood and nutrition quality scores were 17.7 (out of 70), 13.8 (out of 35), and 9 (out of 21), respectively. Univariable regression analysis showed a significant association between sleep quality, mood status, and disease duration, with patients’ status in terms of self-rated wellness and health. In multivariable linear regression, sleep quality was the only significant predictive variable associated with self-rated wellness and health.

**Conclusion:**

Sleep quality was the most important factor associated with low self-rated wellness and health status in patients with Behcet’s disease.

## Background

Chronic diseases have been considered as one of the most challenging burdens in healthcare systems regarding their lifelong duration and incurable course [1, 2]. On this basis, patients with chronic disease are capable of suffering from severe physical, psychological, and psychosocial impairments as the consequences of their illness, which reduces patient’s self-reliance and quality of life [3, 4]. Behcet’s disease (BD) is a chronic multisystemic inflammation with unknown etiology that results in generalized and relapsing clinical manifestations [5]. The majority of clinical signs and symptoms in patients with BD include involvement of mucocutaneous, urogenital, locomotor, ocular, neurological, gastrointestinal, respiratory, and vascular systems [6–8]. Therefore, disease progression contributes to impaired levels of patients’ physical and mental functions that provide severe harmful and destructive impacts on patients’ quality of life [9, 10].

On the other hand, several lifestyle factors might influence the onset, disease course, and the severity of inflammation in patients suffering from chronic and inflammatory disorders [9, 10]. According to the literature, the most important ones are fast foods, omega 3, physical activity, smoking, depression, and sleep patterns [11–20]. Thus, it can be estimated that there is a strong correlation between patients’ habits and lifestyle, and patients’ quality of life, which is reduced or affected through the disease activity and manifestations [21, 22]. With due attention to the recent updates, several studies have suggested a strong correlation between Behcet’s disease and sleep quality of the individuals [23–28]. Furthermore, it has been claimed that the mental health status could be significantly altered in patients suffering from Behcet’s disease; some studies have demonstrated the high frequency of depression and anxiety in these patients [25, 29]. Similarly, smoking has been shown to cause adverse events in means of clinical symptoms and signs of Behcet’s disease [30].

To date, there are limited information regarding lifestyle factors and Behcet’s disease from our country. Therefore, the present study aimed to evaluate the association of essential lifestyle factors and mood status with self-rated wellness and health status in patients with Behcet’s disease.

## Methods

In the current cross-sectional study carried out in Shahid Beheshti hospital, Qom, Iran, 52 patients with a history of Behcet’s disease were enrolled, consecutively. The inclusion criteria for the study included age > 18 years, and fulfilment of International Criteria for Behcet’s Disease for the diagnosis of Behçet’s disease [31]. Patients with psychologic disorders and concomitant chronic disorders were excluded. The study protocol was approved by the Ethics Committee of Qom University of Medical Sciences (IR-MUQ-REC-1398-080), and all patients provided informed consent before enrollment.

Demographic information, body mass index (BMI), cigarette smoking, mood status, sleep quality, physical activity levels, nutritional habits, symptoms and signs, laboratory findings along with patient reported outcome (self-rated wellness and health) were collected. Patients disease activity was calculated via the Iranian Behcet’s Diseases Dynamic Activity Measurement score [31], with due attention to eleven clinical manifestations, as follows: oral aphthae (one point for five ulcers), genital ulceration (one point per ulcer), pseudofolliculitis (one point for ten lesions), erythema nodosum (one point for five lesions), arthritis (arthralgia one point, monoarthritis two points, and polyarthritis three points), venous involvement (thrombophlebitis one point, and massive vessel thrombosis two points), intestinal manifestations (three and six points for mild and moderate to severe manifestations, respectively), central nervous system (CNS) manifestations (one point for mild headache, three points for mild CNS involvements, and six points for moderate to severe manifestations), epididymitis (two points), and pathergy (one point).

Patients’ quality of sleep, mood status, and nutritional condition were evaluated using the Mini-Sleep Questionnaire, Gallup Well-being index, and Gallup Diet Questionnaire, respectively [32–34]. Patients were requested to answer 22 questions provided in the questionnaires; ten questions evaluated sleep quality, five questions assessed mood status, and three questions were aimed to evaluate the nutritional condition. For each question, the frequency of each event ranged from 0 (the complaint never happened) to 7 (patient had the complaints during every day of the week). The overall score calculated by accumulating the scores obtained from questions in each part. A higher grade in each section of the questionnaire demonstrated a worse quality. Physical activity was inquired based on a modified question from Brunel lifestyle physical activity questionnaire [35]. Furthermore, due to the undeniable role of smoking on patients’ lifestyle and quality of life, smoking status was questioned at the end of lifestyle questions [36]. Finally, self-rated wellness and health was included, which represented 20 as the healthiest state and 0 as the unhealthiest [37]. All the questions divided with due attention to their evaluating section, are shown in Table 1.

**Table 1:** Lifestyle questionnaire and the calculating method of corresponding scores

Quality of life measures questionnaire		
Lifestyle factors	Question	Days per week
**Sleep quality**	How many days per week do you have difficulties falling asleep?	**/7**
	How many days per week do you wake up too early?	**/7**
	How many days per week do you use Hypnotic medications (sleep aids)?	**/7**
	How many days per week do you fall asleep during the day?	**/7**
	How many days per week do you feel tired upon waking up in the morning?	**/7**
	How many days per week do you snore?	**/7**
	How many days per week do you experience mid-sleep awakenings?	**/7**
	How many days per week do you experience headaches on awakening?	**/7**
	How many days per week do you experience excessive daytime sleepiness?	**/7**
	How many days per week do you experience excessive movement during sleep?	**/7**
**Total score of sleep quality out of 70**		**/70**
**Mood**	How many days per week do you experience no energy to get things done?	**/7**
	How many days per week do you experience sadness?	**/7**
	How many days per week do you experience worry?	**/7**
	How many days per week do you experience anger?	**/7**
	How many days per week do you experience physical pain?	**/7**
**Total score of mood status out of 35**		**/35**
**Nutrition**	How many days per week do you eat fast food?	**/7**
	How many days per week do you eat fish/omega 3?	**/7**
	How many days per week do you eat 4–5 servings of fruits/vegetables?	**/7**
**Total score of nutrition out of 21**		**/21**
**Physical activity**	How many days per week in a normal week don’t you engage in at least 30- min pre-planned physical activity?	**/7**
**Smoking behavior**	Do you smoke?	
	If yes, how many cigarettes do you smoke per day?	
**Self-rated wellness & health**	How much do you rate your wellness and health out of 20; 20 being the healthiest and 0 being the unhealthiest?	**/20**

### Statistical analysis

Descriptive analyses were conducted to picture the frequency distribution of demographics, lifestyle factors, symptoms, signs and laboratory data. Self-rated wellness and health was a continuous variable. Shapiro-Wilk test was used to assess whether the continuous variables were normally distributed. The frequency distributions of symptoms, signs, and laboratory findings were compared between males and females by chi-square test. Age, disease duration, BMI and smoking rate were compared between males and females by t-test/Mann-Whitney test. Univariable association of every variable and self-rated wellness and health status was evaluated through linear regression analysis. The association of self-rated wellness and health status and lifestyle factors was evaluated by multivariable linear regression model, adjusted for age, sex, BMI, major symptoms and signs and laboratory findings. In order to identify the useful subset of the predictors and reduce the multicollinearity problem and to resolve the overfitting problem, backward elimination process was used. Generalized additive model (GAM) was used to draw the possible non-linear association of self-rated wellness and health status and independent continuous variables. Data analyses were conducted using SPSS program (SPSS version 26, Chicago, IL) and R. *P* value less than 0.05 was considered significant.

## Results

Twenty-one females and 31 males were enrolled. There was no significant difference between genders in terms of age, disease duration, symptoms and signs, and most laboratory findings (Supplementary Table). Nevertheless, female patients had a mean blood urea nitrogen level of 12.5 mg/dL, which was significantly lower than that, 16 mg/dl, in male patients. Furthermore, the mean BMI and mood score were significantly higher in females vs. males (29 vs. 26 kg/m^2^, respectively, for BMI, and 17.6 vs. 11.3, respectively, for mood score). In terms of smoking, men had a significantly higher smoking rate in comparison to women (3.3 vs. 0.5 pack-year, respectively). Oral aphthous, genital aphthous, past or present ocular involvement, pathergy, skin involvement, vascular involvement, and CNS involvement were observed in 100, 52, 92, 36.5, 9.5, 4 and 0% of patients, respectively. Patients’ characteristics and clinical data are summarized in Table 2.

**Table 2:** Demographics, Behcet’s disease and lifestyle characteristics of study sample (SD = Standard Deviation)

Patients’ characteristics	Mean	SD	Minimum	Maximum
**Age, years**	43.94	11.31	23	68
**BMI, kg/m**^**2**^	27.68	3.99	19.59	35.70
**Disease duration, years**	12.11	9.68	1	35
**IBDAAM**	19.37	17.16	0	64
**Eye IBDAAM**	18.92	17.45	0	64
**How many days per week, do you have or experience the followings?**				
**Difficulty falling asleep**	2.33	2.99	0	7
**Too early wake up**	2.81	3.02	0	7
**Hypnotic medications use**	0.62	1.83	0	7
**Falling asleep during the day**	0.56	1.27	0	7
**Tired feeling upon waking**	2.44	2.81	0	7
**Snoring**	2.48	2.79	0	7
**Mid-sleep awakenings**	1.56	2.43	0	7
**Headache upon waking**	1.02	1.99	0	7
**Excessive daytime sleepiness**	2.31	2.76	0	7
**Excessive movement during sleep**	1.56	2.55	0	7
**Total sleep score**	17.67	10.17	0	49
**Lack of energy**	2.29	2.56	0	7
**Sadness**	2.92	2.39	0	7
**Worry**	2.94	2.70	0	7
**Anger**	2.63	2.14	0	7
**Physical pain**	3.04	3.06	0	7
**Total mood status score**	13.83	8.34	1	32
**Fast food meals**	0.44	0.72	0	3
**No fish/omega 3**	2.87	2.46	0	7
**Less than 4/5 servings of fruits & vegetables**	5.67	1.87	0	7
**Total nutrition score**	8.98	3.37	0	14
**No 30-min physical activity**	3.42	3.19	0	7
**Self-rated wellness and health score**	14.60	3.82	0	20

Based on the patients’ report on lifestyle factors according to the questionnaire outcomes, there was no significant difference between male and female patients, while comparing the sleep quality, physical activity, nutritional status, and self-rated wellness and health status. However, females had a significantly higher mood scores in comparison to that of male participants, which proved a worse mood status in women (17.6 vs. 11.3, respectively).

Mean (SD) self-rated wellness and health was 14.6 (3.83) out of 20. Table 3 demonstrates the univariable association of every variable with self-rated wellness and health. Only the following three variables had a significant univariable association with self-rated wellness and health: sleep quality, mood status, and disease duration. When all variables were entered in multivariable linear regression model, only sleep quality remained significant, and mood status was kept in the model by the backward elimination process.

**Table 3:** Linear regression analysis showing univariable and multivariable associations of predictors of self-rated wellness and health (Adjusted *R*^*2*^ = 0.226 and *P* = 0.001)^a^

Predictors	Univariate association		Multivariable model	
	β (95% CI)	***P***	β (95% CI)	***P***
**Age, years**	−0.03 (− 0.13–0.06)	0.50	Removed By Backward Elimination	
**Sex**	0.60 (−1.58–2.78)	0.60		
**Nutritional score**	0.70 (−0.25–0.39)	0.40		
**Mood status score**	−0.18 (− 0.30 - -0.06)	**0.003**	− 0.12 (− 0.24–0.01)	0.07
**Sleep quality score**	−0.17 (− 0.26 - -0.07)	**0.001**	−0.13 (− 0.23 - -0.02)	**0.02**
**Smoking rate, pack-years**	0.08 (−0.13–0.30)	0.40	Removed By Backward Elimination	
**No 30-minute physical activity**	−0.016 (− 0.35–0.32)	0.90		
**BMI, kg/m**^**2**^	−0.07 (− 0.35–0.19)	0.60		
**Disease duration, years**	−0.11 (− 0.21 - -0.003)	**0.04**		
**Genital aphthous**	− 1.39 (− 3.5–0.72)	0.19		
**Ocular involvement**	2.00 (− 1.99–5.99)	0.30		
**Skin involvement**	0.98 (−3.04–4.99)	0.60		
**CRP**	−3.67 (− 11.4–4.08)	0.40		
**Hb level, mg/dl**	0.33 (− 0.42–1.07)	0.40		
**Platelet/Lymphocyte ratio**	− 0.03 (− 0.17–0.11)	0.70		
**Neutrophil/Lymphocyte ratio**	−0.04 (− 0.41–0.34)	0.80		

^a^The dependent variable in this backward linear regression was self-rated wellness and health status. All other variables in Table 3 were considered as independent variables

**Fig. 1:**
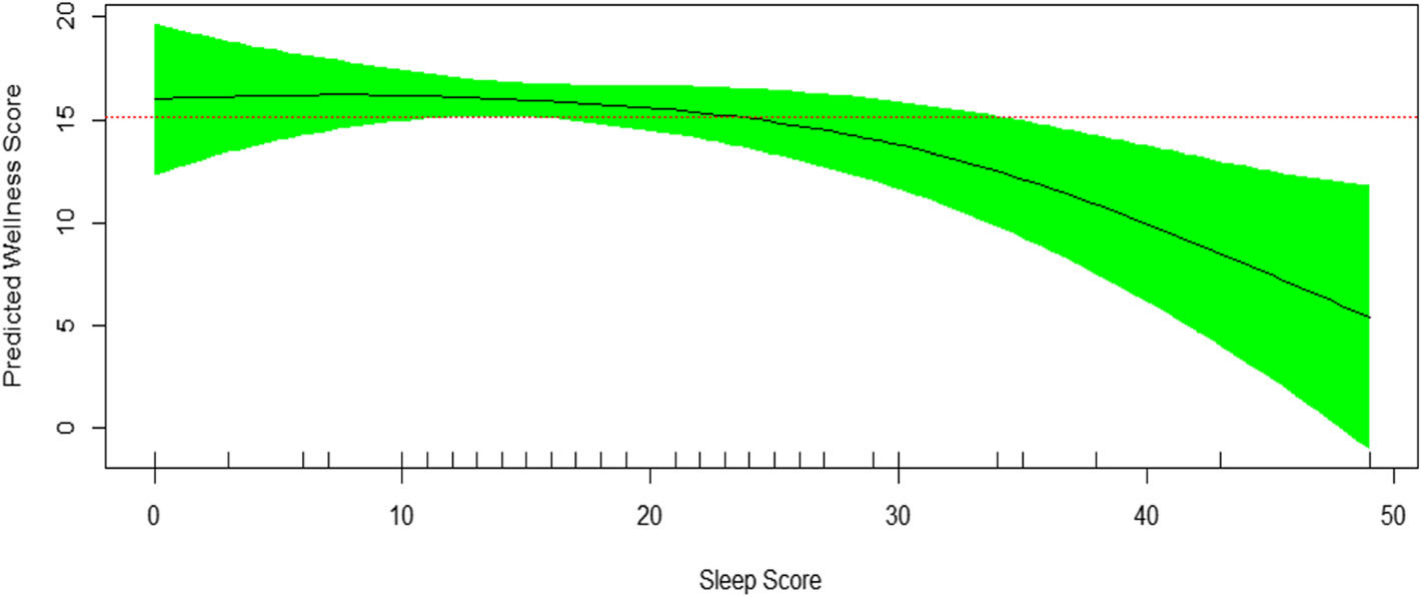
Sleep quality and its association with self-rated wellness and health status (EDF=2.31, R-square=0.21, N=52, *p* value=0.03)

Figure 1 is prepared based on multivariable GAM model. It showed the non-linear association of sleep quality and self-rated wellness and health status. In the current study, the employed questionnaire indicated the score of 70 as the worst sleep quality. Figure 1 showed that a sleep quality score of more than 25 was associated with an obvious decrease in self-rated wellness and health status. Among 10 sleep quality questions, patients showed higher disruptions in five domains of sleep quality as follows: difficulty falling asleep, waking up too early, snoring, excessive daytime sleepiness and feeling tired upon waking up in the morning. Frequency distribution of each of these five domains, happening for more than three nights in a usual week, were 33, 33, 29, 29 and 27%, respectively.

## Discussion

Recently, the quality of life and lifestyle factors in patients suffering from chronic diseases has become a topic of debate concerning their inevitable consequences and influences on several aspects of patients’ physical and mental health through a lifelong disease course [38–40]. The current study evaluated the association of self-rated wellness and health status, as the main patient reported outcome, with lifestyle factors in patients with Behcet’s disease. The final multivariable regression model was adjusted for demographics, smoking habits, nutrition, sleep, physical activity, primary symptoms and signs, and laboratory findings. Sleep quality emerged as the single most important factor associated with self-rated wellness and health status in patients with Behcet’s disease. The presented non-linear plot showed a better quantification of this relationship. According to the plot, if only one-third of sleep quality was disrupted, the patients with Behcet’s disease might begin to feel significantly lower wellness and health status. The association of sleep quality and Behcet’s disease has been shown by previous studies in the literature [23–28]. It seems some aspects of sleep quality are more important than the other ones. Difficulty falling asleep, waking up too early, snoring, excessive daytime sleepiness, and feeling tired upon waking up in the morning were the top sleep disruptions in our patients. The possible mechanism of sleep and inflammatory disorders may come from an imbalance in crucial stages of sleep. Too little deep sleep (stage 3) and too much REM sleep cause hormonal imbalance affecting the level of inflammation [17, 41].

In addition to sleep, mood status was almost significantly associated with self-rated wellness and health status. This association has been shown in Behcet’s disease [24]. The direct therapeutic effects of mood stabilization in improving the outcome in few autoimmune disorders have been demonstrated too [42]. In terms of quality of life and lifestyle assessment in patients with Behcet’s disease, previous studies were mainly aimed at establishing a correlation between patients’ complaints and clinical manifestations and the severity of impairment in quality of life [43–46]. In a study by Khabbazi et al., patients with Behcet’s disease had impaired levels of quality of life, which was in strong correlation with disease severity [47]. Furthermore, the authors suggested genital ulcers as well as the involvement of eye and CNS as the leading causes of impairment in patients’ quality of life. Besides, in another study by Guler et al., oral ulcers and skin lesions were two further clinical manifestations of Behcet’s disease that altered the patients’ physical, social, and emotional functioning and led to impaired quality of life [48]. However, our results showed no significant correspondence between Behcet’s disease patients’ wellness and health status and clinical manifestations, including ocular involvement, genital aphthous, and dermal lesions. But disease duration was negatively associated with patients’ wellness and health status. Thus, it can be hypothesized that prolonged disease duration in chronic illnesses, regardless of the disease type, may result in lower status of self-rated wellness and health. On this basis, we believe the duration of the disease can be considered as the cofounding factor for evaluation of the clinical symptoms and signs effect on patients’ wellness and health status. On the other hand, there is a significant diversity in terms of employed questionnaires for assessment of the quality of life in patients with Behcet’s disease, which might explain part of the controversy of the outcomes among different studies in the literature. Therefore, the designation of a particular and unique questionnaire to evaluate the quality of life in patients with Behcet’s disease is of serious importance, in order to identify and manage the affecting factors.

The main strength of the current survey was the prospective enrollment of patients. Another advantage was the measurement of important lifestyle factors such as nutrition and physical activity along with sleep quality and smoking habit. Also, for the final model, major symptoms, signs, and laboratory findings were considered to draw a better picture of the association between self-rated wellness and health and lifestyle factors. However, our study was of some limitations, as follows: First, a relatively small number of patients agreed to participate in the study by answering the several questions provided in the questionnaire, due to a high number of required information and details. Second, other factors, such as socioeconomic status, might serve as a relevant confounding factor for the evaluation of the lifestyle and quality of life. Third, the cross-sectional design did not allow establishing a cause and effect association between self-rated wellness and health status and sleep quality.

## Conclusion

In conclusion, sleep quality was shown as the single most important index of low self-rated wellness and health status in patients with Behcet’s disease. The non-linear association of sleep quality and self-rated wellness and health status by multivariable GAM model indicated that if only one-third of sleep quality was disrupted, the patients with Behcet’s disease might significantly feel lower wellness and health status.

## Data Availability

Data are available upon request through corresponding author.

## Abbreviations

BD: Behcet’s disease
CNS: Central Nervous System
GAM: Generalized Additive Models
